# Genomes of diverse isolates of the marine cyanobacterium *Prochlorococcus*

**DOI:** 10.1038/sdata.2014.34

**Published:** 2014-09-30

**Authors:** Steven J. Biller, Paul M. Berube, Jessie W. Berta-Thompson, Libusha Kelly, Sara E. Roggensack, Lana Awad, Kathryn H. Roache-Johnson, Huiming Ding, Stephen J. Giovannoni, Gabrielle Rocap, Lisa R. Moore, Sallie W. Chisholm

**Affiliations:** 1 Department of Civil and Environmental Engineering, Massachusetts Institute of Technology, Cambridge, Massachusetts, USA; 2 Microbiology Graduate Program, Massachusetts Institute of Technology, Cambridge, Massachusetts, USA; 3 Department of Biological Sciences, University of Southern Maine, Portland, Maine, USA; 4 Department of Biology, Massachusetts Institute of Technology, Cambridge, Massachusetts, USA; 5 Department of Microbiology, Oregon State University, Corvallis, Oregon, USA; 6 School of Oceanography, Center for Environmental Genomics, University of Washington, Seattle, Washington, USA

## Abstract

The marine cyanobacterium *Prochlorococcus* is the numerically dominant photosynthetic organism in the oligotrophic oceans, and a model system in marine microbial ecology. Here we report 27 new whole genome sequences (2 complete and closed; 25 of draft quality) of cultured isolates, representing five major phylogenetic clades of *Prochlorococcus*. The sequenced strains were isolated from diverse regions of the oceans, facilitating studies of the drivers of microbial diversity—both in the lab and in the field. To improve the utility of these genomes for comparative genomics, we also define pre-computed clusters of orthologous groups of proteins (COGs), indicating how genes are distributed among these and other publicly available *Prochlorococcus* genomes. These data represent a significant expansion of *Prochlorococcus* reference genomes that are useful for numerous applications in microbial ecology, evolution and oceanography.

## Background & Summary

**Figure 1:**
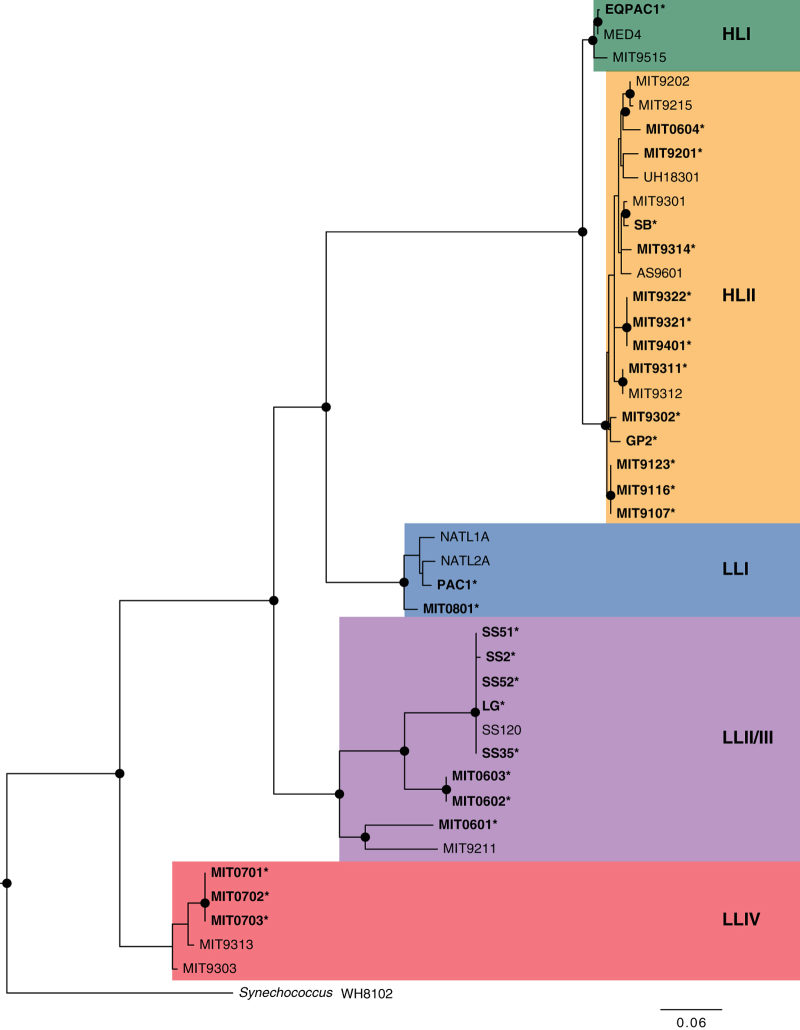
*Prochlorococcus* strains sequenced in this work. ITS-based phylogeny of the strains included in this data set (names in bold, with *) in relation to previously sequenced *Prochlorococcus*. Phylogenetic clade affiliation^4,6^ is indicated at right; closed circles indicate nodes with bootstrap support >75%. HL—High light adapted; LL—Low light adapted, as determined by physiological studies of some of the isolates^3,5,7^.

As the smallest (<1 μm diameter) and most abundant (3×10^27^ cells) photosynthetic organism on the planet^1^, *Prochlorococcus* has a unique status in the microbial world. This unicellular marine cyanobacterium is found throughout the euphotic zone of the open ocean between ~45**°**N and 40**°**S, where it carries out a notable fraction of global photosynthesis^1,^ ^2^. The group, which would be considered a single microbial ‘species’ by the traditional measure of >97% 16S rRNA similarity, is composed of multiple phylogenetically distinct clades (Figure 1) (as defined by either rRNA internal transcribed spacer (ITS)^3^ or whole-genome sequences^4^) which are physiologically distinct. Adaptations for optimal growth at different light intensities differentiate deeply branching groups of *Prochlorococcus* into high light (HL) and low light (LL) adapted clades^3,5–8^.

*Prochlorococcus* have the smallest genomes of any known free-living photosynthetic cell, ranging from ~1.6 to 2.7 Mbp^4^. While they all share a core set of genes present in all strains, there exists remarkable diversity in gene content among isolates. The group has an ‘open’ pan-genome, i.e. each newly sequenced genome typically contains many new genes never before seen in *Prochlorococcus* ^4^. Given the abundance of *Prochlorococcus*, studies of their genomic and metagenomic features have provided numerous insights into features of ocean ecosystems^9–17^. In addition, the group has proven to be a valuable system for studying microbial evolution^18,19^, genome streamlining^20,21^, and the relationship between genotypic, phenotypic and ecological variation in marine populations^3,7,22^. Since *Prochlorococcus* is abundant in surface waters, these reference genomes have also been extremely valuable for interpreting marine metagenomic and metatranscriptomic datasets^14,23–28^.

To advance our understanding of *Prochlorococcus* genetic diversity, we sequenced the genomes of 27 *Prochlorococcus* strains from a variety of ocean environments. The strains sequenced included both previously reported strains as well as eight new isolates (Table 1). The newly isolated strains come from ocean regions that previously only had few or no cultured representatives and substantially expand the number of cultured *Prochlorococcus* available for five major clades. These results demonstrate the applicability of high-throughput dilution-to-extinction cultivation approaches^29^ to *Prochlorococcus*.

The genome sequences reported here represent a notable increase in the number of genome sequences available from the major phylogenetic clades with existing cultured representatives. While many genomes differed greatly in gene content, other sets are very closely related and differ primarily by single nucleotide polymorphisms (e.g., LG, SS2, SS35, SS51, SS52, SS120; and MIT0701, MIT0702, and MIT0703). Thus, this dataset encompasses a broad range of pairwise genomic diversity among *Prochlorococcus* strains.

Most genomes were sequenced to draft status; two were closed (Table 2). We used two annotation methods to identify the potential functions of genes in the genomes. Genes were first called and annotated by the RAST pipeline^30^. To expand on these predictions—especially for the myriad genes of unknown function—we also derived annotations from an independent pipeline, Argot2^31^. To facilitate the utility of these genomes for comparative genomics and evolutionary studies, we define a set of pre-computed orthologous gene clusters for *Prochlorococcus.* All cluster data are supplied in this data set (Data Citation 1 and Data Citation 2).

These genomes should be useful to researchers interested in many aspects of marine microbial ecology and evolution. Since the genomes are from cultured isolates, hypotheses generated from these data can be tested in laboratory experiments. The genomes will also greatly facilitate the interpretation of transcriptomic and proteomic studies, as well as meta-‘omic’ data from field studies where *Prochlorococcus* is a dominant phototroph.

## Methods

### Culturing and strain isolations

Many of the strains sequenced have been previously described^3,5,6,32–36^ (Table 1); 8 are reported here for the first time. All cultures were unialgal; this was initially determined crudely by flow cytometry profiles, and then more specifically by confirming the presence of only one cyanobacterial 16S rRNA ITS sequence in the culture. All cultures except SB and MIT0604 contained heterotrophic bacteria. Cultures were maintained in acid-washed glassware in Pro99 media^37^ prepared with 0.2 μm filtered, autoclaved seawater collected from Vineyard Sound, MA or the Sargasso Sea under either a 14:10 light:dark cycle at 24 °C or constant light flux at 21 °C. Light levels were 30–40 μmol Q m^−2^ s^−1^ for high-light adapted strains, and 10–20 μmol Q m^−2^ s^−1^ for low-light adapted strains.

MIT0601, MIT0602, MIT0603, and MIT0604 were derived from enrichment cultures initiated with seawater obtained from the North Pacific Ocean at Station ALOHA (22.75°N, 158°W) on Hawai’i Ocean Time-series (HOT) cruise 181. The seawater was amended with nitrogen, phosphorous and trace metals (PRO2 nutrient additions^37^, except all nitrogen sources were replaced by 0.217 mM sodium nitrate).

Strains MIT0701, MIT0702, and MIT0703 were isolated from the South Atlantic (CoFeMUG cruise KN192-05, station 13, 13.45 °S, 0.04 °W) at 150 m using a high throughput culturing method^29^ adapted for phototrophs. The seawater used for isolations was first filtered through a 1 μm filter with no amendments and kept in the dark at 18–20 °C for 21 days. The total red fluorescing phytoplankton population (1×10^5^ cells ml^−1^ determined with a Guava EasyCyte flow cytometer) was diluted in PRO3V media^37^ made with the same South Atlantic water that had been filtered through a 0.1 μm Supor 142 mm filter, then autoclaved to sterilize. This media contained 100 μM NH_4_Cl, 10 μM NaH_2_PO_4_, PRO2 trace metals^37^ and f/2 vitamins (0.1 μg l^−1^ cyanocobalamin, 20 g l^−1^ thiamin and 1 μg l^−1^ biotin^38,39^). Ten cells were dispensed into 1 ml volumes in a 48-well polystyrene multiwell culture plate and incubated at 20 °C in ~20 μmol Q m^−2^ s^−1^ (14:10 light:dark) for 2 months.

MIT0801 was isolated in a similar manner, but from seawater obtained from 40 m depth at the Bermuda Atlantic Time-series station (BATS; 31.67 °N, 64.16 °W) that had been sitting in the dark for 5 days. The same PRO3V media recipe was made with 0.1 μm filtered and autoclaved BATS seawater, and 2.5 cells (on average) were dispensed in 5 ml volume in Teflon plates (prepared as described^29^). Cells were detected within 1 month of enrichment.

### DNA sequencing and assembly

Genomes were sequenced from genomic DNA collected from 20 ml laboratory cultures. Cells were collected by centrifugation (10,000*g*, 10 min), the pellet transferred into a 2 ml tube and frozen at −80 °C. Genomic DNA was isolated using the QIAamp DNA mini kit (Qiagen). 2 μg of DNA was then used to construct an Illumina sequencing library as previously described^40^, except that the bead:sample ratios in the double solid phase reversible immobilization (dSPRI) size-selection step were 0.7 followed by 0.15, resulting in fragments with an average size of ~340 bp (range: 200–600 bp). PAC1 and EQPAC1 libraries were constructed using dSPRI bead:sample ratios of 0.9 followed by 0.21, yielding an average size of ~220 bp. DNA libraries were sequenced on an Illumina GAIIx, producing 200+200 nt paired reads, at the MIT BioMicro Center. An average of 1.6 million paired-end reads were obtained for each genome.

Low quality regions of sequencing data were removed from the raw Illumina data using quality_trim (V3.2, from the CLC Assembly Cell package; CLC bio) with default settings (at least 50% of the read must be of a minimum quality of 20). Paired-end reads were overlapped using the SHE-RA algorithm^41^, keeping any resulting overlapping sequences with an overlap score >0.5. For all genomes except PAC1 and EQPAC1, the overlapped reads, as well as the trimmed paired-end reads that did not overlap, were assembled using the Newbler assembler (V2.6; 454/Roche) with the following parameters: ‘-e 200 –rip.’ Contigs <1 Kbp were discarded at this stage.

Reads for PAC1 and EQPAC1 were assembled using clc_novo_assemble (V3.2, from the CLC Assembly Cell package; CLC bio) with a minimum contig length of 500 bp and automatic wordsize determination enabled. These initial contigs were searched against a custom database of marine microbial genomes^9^ using BLAST^42^ to identify contigs with a closest match to *Prochlorococcus*. Sequencing reads belonging to the putative *Prochlorococcus* contigs were then identified by mapping the raw sequences to these contigs using clc_ref_asssemble_long (CLC bio). The *Prochlorococcus-*like reads were then re-assembled using clc_novo_assemble using the same parameters as above to produce the final assembly, now largely free of heterotrophic sequences.

MIT0604 and MIT0801 were completed to finished quality with no gaps by directed PCR reactions to sequence contig junctions, combined with Pacific Biosciences long sequencing reads. Contigs were ordered into putative scaffolds based on their similarity to closely related closed *Prochlorococcus* genomes, as determined by Mauve^43^. PCR primers specific to the ends of putatively adjacent contigs were designed and used to amplify the junctions between contigs. Purified PCR products were sequenced by Sanger chemistry at the MGH DNA core facility, and the resulting sequences used to join contigs in Consed^44^. This resulted in a highly improved but still incomplete assembly. To span difficult repeat regions in MIT0801, we obtained long Pacific Biosciences sequences. We obtained DNA from 25 ml cultures using the Epicentre Masterpure kit (Epicentre) and sequenced this at the Yale Center for Genome Analysis. We combined this set of long but low quality reads with the high quality Illumina short reads obtained previously using the PacBioToCA software^45^, to produce assemblies with a reduced number of contigs. These contigs were aligned to the PCR-improved contigs described above, and the final gaps were closed with a small number of additional directed PCR reactions (as described above) using the Geneious sequence analysis package (V6.1, Biomatters), until the genomes were closed.

As most of the *Prochlorococcus* cultures sequenced were known to contain heterotrophs, we identified the most ‘*Prochlorococcus*-like’ contigs from non-axenic cultures by searching each resulting contig against a custom database of sequenced marine microbial genomes^9^ using BLAST^42^. Contigs with a best match to a non-*Prochlorococcus* genome were removed from the assembly. Subsequent examination of these contig sets indicated that a number of shorter sequences (generally <10 kbp) with significant heterotroph-like stretches had passed through the initial filtering steps. To remove these questionable contigs from the assemblies, we manually examined each <10 kbp contig using the RAST annotation server (see below), and only kept those contigs with clear homology to previously sequenced and closed *Prochlorococcus* or *Synechococcus* genomes. Although these filtering steps may have removed a small amount of true *Prochlorococcus* sequence from the final assembly, we considered missing a few genes preferable to misrepresenting heterotroph sequences as *Prochlorococcus*.

Examination of the non-cyanobacterial 16S rRNA genes found within these data indicate that the most abundant heterotrophs in the cultures were members of the *Alteromonadales*, *Flavobacteriales*, *Rhodospirillales*, *Halomonadaceae*, and *Sphingobacteriales*. We have included a separate data file containing all of the assembled contigs—including those from co-cultured heterotrophs—for anyone who is interested (Data File 4).

### Genome annotation

The assembled contigs for each genome were annotated using the RAST server^30^ against FIGfam release 49. Additional functional annotation for all genes called by RAST were generated by the Argot2 server^31^, using default settings.

To confirm the rRNA-based phylogeny of these strains, rRNA ITS sequences were aligned in ARB^46^ and maximum likelihood phylogenies calculated in PhyML version 20120412^47^, using the HKY85 model of nucleotide substitution, a fixed proportion of invariable sites, and non-parametric bootstrap analysis with 100 replicates.

Clusters of orthologous groups of proteins (COGs) were computed, as described elsewhere^48^, on a data set comprised of previously sequenced *Prochlorococcus* and *Synechococcus* strains^4,10,16,17,49–53^, the new *Prochlorococcus* genomes described here, 11 *Prochlorococcus* single-cell genomes^12^ and two consensus metagenomic assemblies^14^ (Data Citation 1). To facilitate comparisons among genomes, we re-annotated 16 previously sequenced *Prochlorococcus* genomes (Table 3) with the RAST pipeline as described above; this ensured that a uniform methodology for gene calling and functional annotation was used. Single cell genomes^12^ were not re-annotated due to difficulties encountered using this pipeline on such fragmented contigs; instead, we utilized the ORFs previously defined in GenBank. Detailed information regarding these updated annotations is provided (Data Citation 1 and Data Citation 2).

Orthologous gene clusters were defined based on reciprocal best blastp scores (with an e-value cutoff of 1e−5); the sequence alignment length had to be at least 75% of the shorter protein, with at least a 35% identity. Additional orthologous genes that did not pass this criterion were added to clusters based on HMM profiles constructed from automated MUSCLE^54^ alignments of orthologous sequences within each cluster using HMMER^55^. The clusters described here are noted as ‘V4’ CyCOGs in the associated Data Records and on the ProPortal website^48^ (Data Citation 1).

## Data Records

The complete dataset is available from the *Prochlorococcus* Portal website (Data Citation 1) and Dryad (Data Citation 2). The 27 *Prochlorococcus* genome sequences have also been deposited at DDBJ/EMBL/GenBank (3, 4, 5, 6, 7, 8, 9, 10, 11, 12, 13, 14, 15, 16, 17, 18, 19, 20, 21, 22, 23, 24, 25, 26, 27, 28, 29) under the accession numbers indicated in Table 2.

### Datasets deposited at Dryad and ProPortal

Sequence, gene annotations, and COG definitions for *Prochlorococcus* genomes.

File 1—Tab-delimited file containing cluster assignments and annotation metadata for genes in the newly sequenced *Prochlorococcus* genomes described in this work, as well as previously published genomes. Columns are as follows:

#### Genome

The *Prochlorococcus* strain where the gene is found.

#### Gene ID

Unique ID for each *Prochlorococcus* gene, of the format ‘P<strain>_####’. Note that, due to the re-annotation of previously published genomes, these names (and the underlying gene boundaries) may not necessarily correspond to those in Genbank.

#### NCBI ID

For the new genome sequences presented here, the systematic NCBI locus_tag identifier for that gene. For previously published genomes, this column contains the corresponding Genbank locus ID (noted as an ‘Alternative locus ID’ for strains MED4, SS120 and MIT9313 in Genbank) from Kettler *et al.* (2007)^4^.

#### V1 CyCOG

Where applicable, the cyanobacterial cluster of orthologous groups of proteins (CyCOG) definition from Kettler *et al.* (2007)^4^.

#### V3 CyCOG

Where applicable, the CyCOG definition from Kelly *et al.* (2013)^56^.

#### V4 CyCOG

Number indicating the CyCOG to which this gene belongs, as defined in this work.

#### RAST annotation

Predicted functional annotation description, as supplied by the RAST annotation pipeline. Note that this text may differ slightly from the annotation in Genbank, due to changes imposed by NCBI annotation formatting guidelines.

#### GO annotation

Gene Ontology categorization for the gene, when available.

#### Argot2 annotation

Functional annotation prediction from the Argot2 pipeline, when available.

File 2 – Full RAST gene/protein sequence and annotation results. ZIP format file archive of individual tab-delimited files. Files are supplied for the new genome sequences presented here, as well as re-annotations of previously published genomes included in the CyCOG definitions. Columns are as follows:

#### contig_id

The name of the sequence contig on which the gene is found.

#### gene_id

The unique Gene ID code for that feature.

#### feature_id

Unique RAST-generated identifier for that feature.

#### type

peg: protein encoding gene; rna: RNA molecule.

#### location

Ordered location code for the position on the genome merging contig_id, start, and stop position.

#### start

Start location on contig, bp.

#### stop

Stop location on contig, bp.

#### strand

Orientation of gene on contig (+: on forward strand; −: on reverse).

#### function

The predicted function of the feature, if known.

#### aliases

Alternative names for the predicted function.

#### figfam

FigFAM membership for that feature.

#### evidence_codes

Code indicating the reason for the annotation. See http://www.nmpdr.org/FIG/wiki/view.cgi/FIG/EvidenceCode for more details.

#### nucleotide_sequence

The nucleotide sequence of the predicted gene.

#### aa_sequence

The protein (amino acid) sequence of the predicted gene.

File 3 – Set of nucleotide FASTA-formatted files containing the new *Prochlorococcus* genome assemblies described in this work.

File 4 – Set of nucleotide FASTA files containing all assembled contigs (>500 bp) from each culture (i.e., both *Prochlorococcus* and heterotrophs) sequenced in this work. Each file contains the set of contigs assembled from the raw sequencing data, before any filtering to separate *Prochlorococcus* from heterotroph contigs. These files are provided for reference, but due to the known heterotroph sequences in these files, they should be used with caution.

File 5 – Set of nucleotide FASTA files containing the predicted nucleotide sequence for all open reading frames (ORFs) in each genome. This file includes ORFs from both the new genomes presented here as well as the re-annotation of previously released *Prochlorococcus* genomes.

File 6 – Set of protein FASTA files containing the predicted amino acid translation for all ORFs in each genome. This file includes ORFs from both the new genomes presented here as well as the re-annotation of previously released *Prochlorococcus* genomes.

## Technical Validation

Phylogenetic analysis of the ITS sequences obtained from these cultured isolate genomes (Figure 1) group these strains into the expected clades^57^ as previously determined from directed sequencing of the ITS sequences^6^. We were only able to obtain a single cyanobacterial ITS sequence from the assembled genome contigs, again consistent with these strains being unialgal. *Prochlorococcus* genome size and %GC content are typically quite similar for strains found within the same ITS-defined clade^4^, and both the draft and closed genomes are consistent with previously sequenced strains for these measures as well (Table 2).

The quality of the genome assemblies was assessed in multiple ways. Re-mapping of the original Illumina sequencing reads to the final assembled contigs showed that the reads were distributed evenly along the length of the assembly, ruling out some categories of major assembly errors (such as duplicated regions). Whole-genome alignments of contigs against closely related closed reference *Prochlorococcus* genomes indicated that the overall gene order of these contigs was broadly consistent with known sequences, indicating that the sequences do not contain obvious chimeras or other artifacts. We also estimated the completeness of the draft genomes by examining the core gene content of the strains, based on the set of genes shared by all closed *Prochlorococcus* genomes. We found that all of the draft genome assemblies contained >98% of the genes universally present in the 13 previously published closed *Prochlorococcus* genomes, indicating that these contigs represent most (or perhaps all) of the genome sequence.

The final closed sequences of the MIT0604 and MIT0801 genomes were verified in two additional ways. First, we compared the experimentally observed PCR product sizes from directed contig joining reactions to the distances predicted from the final assembled sequence to confirm the assembly. Second, we mapped the original (quality trimmed) Illumina sequencing reads against the final assembly. These alignments indicated that the final closed assembly was fully consistent with the original short-read sequence data. In addition, we confirmed that the per-base SNP frequency was not above the expected error frequency.

## Additional information

**How to cite this article:** Biller, S. J. *et al.* Genomes of diverse isolates of the marine cyanobacterium *Prochlorococcus*. *Sci. Data* 1:140034 doi: 10.1038/sdata.2014.34 (2014).

## Supplementary Material



## Figures and Tables

**Table 1:** Origin of the *Prochlorococcus* strains sequenced in this study.

**Strain**	**Alternate Name**	**Ecotype/Clade** ^4,57^	**Isolation location**	**Isolation (Lat/Lon)**	**Isolation depth (m)**	**Isolation date**	**Strain reference**
EQPAC1	RCC278	eMED4/HLI	Equatorial Pacific	0°N 180°W	30		Roscoff Culture Collection
GP2		eMIT9312/HLII	Western Pacific	8°N 136°E	150	Sep-1992	32
MIT0604		eMIT9312/HLII	Station ALOHA/North Pacific	22.75°N 158°W	175	May-2006	This work
MIT9107		eMIT9312/HLII	Tropical Pacific	15°S 135°W	25	8-Aug-1991	33
MIT9116		eMIT9312/HLII	Tropical Pacific	15°S 135°W	25	8-Aug-1991	6
MIT9123		eMIT9312/HLII	Tropical Pacific	15°S 135°W	25	8-Aug-1991	6
MIT9201		eMIT9312/HLII	Tropical Pacific	12°S 145.42°W	Surface	26-Sep-1992	5
MIT9302		eMIT9312/HLII	Sargasso Sea	34.76°N 66.19°W	100	15-Jul-1993	3
MIT9311		eMIT9312/HLII	Gulf stream	37.51°N 64.24°W	135	17-Jul-1993	6
MIT9314		eMIT9312/HLII	Gulf stream	37.51°N 64.24°W	180	17-Jul-1993	6
MIT9321		eMIT9312/HLII	Equatorial Pacific	1°N 92°W	50	12-Nov-1993	6
MIT9322		eMIT9312/HLII	Equatorial Pacific	0.27°N 93°W	Surface	16-Nov-1993	6
MIT9401		eMIT9312/HLII	Sargasso Sea	35.5°N 70.4°W	Surface	May-1994	6
SB		eMIT9312/HLII	Western Pacific	35°N 138.3°E	40	1-Oct-1992	32
MIT0801	HTCC 1603	eNATL/LLI	BATS/Sargasso Sea	31.67°N 64.17°W	40	25-Mar-2008	This work
PAC1		eNATL/LLI	Station ALOHA/North Pacific	22.75°N 158°W	100	1992	34,35
LG		eSS120/LLII,III	Sargasso Sea	28.98°N 64.35°W	120	30-May-1988	36
MIT0601		eMIT9211/LLII,III	Station ALOHA/North Pacific	22.75°N 158°W	125	17-Nov-2006	This work
MIT0602		eSS120/LLII,III	Station ALOHA/North Pacific	22.75°N 158°W	125	17-Nov-2006	This work
MIT0603		eSS120/LLII,III	Station ALOHA/North Pacific	22.75°N 158°W	125	17-Nov-2006	This work
SS2		eSS120/LLII,III	Sargasso Sea	28.98°N 64.35°W	120	30-May-1988	6
SS35		eSS120/LLII,III	Sargasso Sea	28.98°N 64.35°W	120	30-May-1988	6
SS51		eSS120/LLII,III	Sargasso Sea	28.98°N 64.35°W	120	30-May-1988	6
SS52		eSS120/LLII,III	Sargasso Sea	28.98°N 64.35°W	120	30-May-1988	6
MIT0701	HTCC 1600	eMIT9313/LLIV	South Atlantic	13.45°S 0.04°W	150	1-Dec-2007	This work
MIT0702	HTCC 1601	eMIT9313/LLIV	South Atlantic	13.45°S 0.04°W	150	1-Dec-2007	This work
MIT0703	HTCC 1602	eMIT9313/LLIV	South Atlantic	13.45°S 0.04°W	150	1-Dec-2007	This work

**Table 2:** Genome characteristics and assembly statistics.

**Strain**	**Clade** ^4^	**Assembly size (bp)**	**%GC**	**No. contigs**	**N50 (bp)**	**No. coding sequences**	NCBI accession*
EQPAC1	HLI	1,654,739	30.8	8	328,627	1,954	JNAG00000000
GP2	HLII	1,624,310	31.2	11	416,038	1,884	JNAH00000000
MIT0604	HLII	1,780,061	31.2	1	1,780,061	2,085	CP007753
MIT9107	HLII	1,699,937	31.0	13	170,362	1,991	JNAI00000000
MIT9116	HLII	1,685,398	31.0	22	117,620	1,972	JNAJ00000000
MIT9123	HLII	1,697,748	31.0	18	137,374	2,005	JNAK00000000
MIT9201	HLII	1,672,416	31.3	21	145,955	1,989	JNAL00000000
MIT9302	HLII	1,745,343	31.1	17	242,124	2,015	JNAM00000000
MIT9311	HLII	1,711,064	31.2	17	189,094	1,983	JNAN00000000
MIT9314	HLII	1,690,556	31.2	16	221,824	1,990	JNAO00000000
MIT9321	HLII	1,658,664	31.2	10	259,210	1,956	JNAP00000000
MIT9322	HLII	1,657,550	31.2	11	367,597	1,959	JNAQ00000000
MIT9401	HLII	1,666,808	31.2	17	110,519	1,972	JNAR00000000
SB	HLII	1,669,823	31.5	4	1,237,529	1,933	JNAS00000000
MIT0801	LLI	1,929,203	34.9	1	1,929,203	2,287	CP007754
PAC1	LLI	1,841,163	35.1	20	182,484	2,264	JNAX00000000
LG	LLII,III	1,754,063	36.4	14	326,623	1,973	JNAT00000000
MIT0601	LLII,III	1,707,342	37.0	6	547,047	1,934	JNAU00000000
MIT0602	LLII,III	1,750,918	36.3	9	511,704	1,998	JNAV00000000
MIT0603	LLII,III	1,752,482	36.3	7	434,668	2,015	JNAW00000000
SS2	LLII,III	1,752,772	36.4	19	187,268	1,989	JNAY00000000
SS35	LLII,III	1,751,015	36.4	9	446,270	1,977	JNAZ00000000
SS51	LLII,III	1,746,977	36.4	12	232,789	1,974	JNBD00000000
SS52	LLII,III	1,754,053	36.4	22	124,224	1,987	JNBE00000000
MIT0701	LLIV	2,592,571	50.6	53	84,463	3,079	JNBA00000000
MIT0702	LLIV	2,583,057	50.6	61	76,101	3,066	JNBB00000000
MIT0703	LLIV	2,575,057	50.6	61	81,186	3,054	JNBC00000000
*For the Whole Genome Shotgun projects deposited at DDBJ/EMBL/GenBank: the version described in this paper is version JN**01000000.							

**Table 3:** Previously sequenced *Prochlorococcus* genomes included in the cyanobacterial clusters of orthologous groups of proteins (CyCOG) definitions.

**Name**	**Genome source**	**Clade**	**Assembly size (bp)**	**%GC**	No. coding sequences*	**NCBI accession**	**Sequence reference**
MED4	Cultured isolate	HLI	1,657,990	30.8	1,959	BX548174	10
MIT9515	Cultured isolate	HLI	1,704,176	30.8	1,951	CP000552	4
AS9601	Cultured isolate	HLII	1,669,886	31.3	1,944	CP000551	4
MIT9202	Cultured isolate	HLII	1,691,453	31.1	2,000	DS999537	49
MIT9215	Cultured isolate	HLII	1,738,790	31.1	2,035	CP000825	4
MIT9301	Cultured isolate	HLII	1,641,879	31.3	1,925	CP000576	4
MIT9312	Cultured isolate	HLII	1,709,204	31.2	1,982	CP000111	16
UH18301	Cultured isolate	HLII	1,654,648	31.2	1,947	PRJNA47033	50
W6	Single cell amplified genome	HLII	385,307	31.3	646	ALPK00000000	12
HNLC2	Metagenomic assembly	HLIII	1,484,494	30.3	1,701	GL947595	14
W3	Single cell amplified genome	HLIII	339,045	30.7	529	ALPC00000000	12
W5	Single cell amplified genome	HLIII	99,467	29.8	212	ALPL00000000	12
W7	Single cell amplified genome	HLIII	905,221	30.7	989	ALPE00000000	12
W8	Single cell amplified genome	HLIII	841,756	31.4	917	ALPF00000000	12
W9	Single cell amplified genome	HLIII	420,150	30.7	638	ALPG00000000	12
HNLC1	Metagenomic assembly	HLIV	1,569,623	29.8	1,830	GL947594	14
W10	Single cell amplified genome	HLIV	561,998	30.8	892	ALPH00000000	12
W11	Single cell amplified genome	HLIV	766,829	30.6	929	ALPI00000000	12
W12	Single cell amplified genome	HLIV	423,437	29.6	602	ALPJ00000000	12
W2	Single cell amplified genome	HLIV	1,266,767	30.5	1,374	ALPB00000000	12
W4	Single cell amplified genome	HLIV	765,485	29.9	819	ALPD00000000	12
NATL1A	Cultured isolate	LLI	1,864,731	35.0	2,242	CP000553	4
NATL2A	Cultured isolate	LLI	1,842,899	35.1	2,194	CP000095	4
MIT9211	Cultured isolate	LLII,III	1,688,963	38.0	1,943	CP000878	4
SS120	Cultured isolate	LLII,III	1,751,080	36.4	1,973	AE017126	17
MIT9303	Cultured isolate	LLIV	2,682,675	50.0	3,253	CP000554	4
MIT9313	Cultured isolate	LLIV	2,410,873	50.7	2,993	BX548175	10
*For the cultured isolate and metagenomic assembly genomes, this value represents the number of coding sequences as predicted in this study using the RAST pipeline; these values may differ from those previously published for this reason. Re-annotation data is included in this dataset (Data Citation 1 and Data Citation 2).							
